# Chronic psychological stress activates BMP4-dependent extramedullary erythropoiesis

**DOI:** 10.1111/jcmm.12167

**Published:** 2013-11-27

**Authors:** Sanja Vignjević, Mirela Budeč, Dragana Marković, Dragoslava Đikić, Olivera Mitrović, Slavko Mojsilović, Sanja Vranješ Đurić, Vesna Koko, Bojana Beleslin Čokić, Vladan Čokić, Gordana Jovčić

**Affiliations:** a Institute for Medical Research, University of BelgradeBelgrade, Serbia; b Institute for Nuclear Sciences “Vinča”, University of BelgradeBelgrade, Serbia; c Clinic for Endocrinology, Diabetes and Metabolic Diseases Genetic Laboratory, Clinical Center of SerbiaBelgrade, Serbia

**Keywords:** stress, erythropoiesis, BMP4, spleen

## Abstract

Psychological stress affects different physiological processes including haematopoiesis. However, erythropoietic effects of chronic psychological stress remain largely unknown. The adult spleen contains a distinct microenvironment favourable for rapid expansion of erythroid progenitors in response to stressful stimuli, and emerging evidence suggests that inappropriate activation of stress erythropoiesis may predispose to leukaemic transformation. We used a mouse model to study the influence of chronic psychological stress on erythropoiesis in the spleen and to investigate potential mediators of observed effects. Adult mice were subjected to 2 hrs daily restraint stress for 7 or 14 consecutive days. Our results showed that chronic exposure to restraint stress decreased the concentration of haemoglobin in the blood, elevated circulating levels of erythropoietin and corticosterone, and resulted in markedly increased number of erythroid progenitors and precursors in the spleen. Western blot analysis revealed significantly decreased expression of both erythropoietin receptor and glucocorticoid receptor in the spleen of restrained mice. Furthermore, chronic stress enhanced the expression of stem cell factor receptor in the red pulp. Moreover, chronically stressed animals exhibited significantly increased expression of bone morphogenetic protein 4 (BMP4) in the red pulp as well as substantially enhanced mRNA expression levels of its receptors in the spleen. These findings demonstrate for the first time that chronic psychological stress activates BMP4-dependent extramedullary erythropoiesis and leads to the prolonged activation of stress erythropoiesis pathways. Prolonged activation of these pathways along with an excessive production of immature erythroid cells may predispose chronically stressed subjects to a higher risk of leukaemic transformation.

## Introduction

Stress is an integral part of daily life 1. Different psychological stressors evoke physiological changes that perturb homeostasis, and accumulating evidence suggests that chronic psychological stress promotes cancer genesis and progression 2,3.

Restraint stress, an experimental model of psychological stress 5, affects different physiological processes including haematopoiesis 6. Accordingly, Sherstoboev and Minakova showed an expansion of erythroid progenitors in the bone marrow during chronic stress 7. However, unlike steady-state erythropoiesis that occurs predominantly in the bone marrow, under various conditions, collectively referred to as stress erythropoiesis (SE), the adult spleen becomes a major site of red blood cell production 8–9. The spleen has also been recognized as a major site of extramedullary haematopoiesis in certain pathological conditions, such as myeloproliferative neoplasms 10. Furthermore, several lines of evidence indicate that erythropoiesis in the adult bone marrow is primarily homeostatic and presumably unable to cope with an excessive demand for red blood cells 11–12. In contrast, the adult spleen contains a unique microenvironment that is favourable for rapid expansion of erythroid progenitors in response to stressful stimuli 13,14.

Aside from being found in different organs, homeostatic and SE also differ in the particular signals to which they respond 11. The signals for SE are integrated through the co-operative interactions among erythropoietin (EPO), glucocorticoids, stem cell factor (SCF) and bone morphogenetic protein 4 (BMP4) 16–17. Recently, Paulson *et al*. have demonstrated a new model for SE in which BMP4 drives the expansion of specialized stress erythroid progenitors in the spleen 18. In addition, growing evidence suggests that inappropriate activation of these SE pathways may predispose to leukaemic transformation 8–19.

Stress erythropoiesis has been studied mainly in murine models of acute anaemia 13–20. However, the erythropoietic effects of chronic psychological stress remain largely unknown. Therefore, the purpose of this study was: (*i*) to examine the influence of chronic restraint stress on erythropoiesis in the murine spleen and (*ii*) to investigate the involvement of erythropoietin receptor (EPOR), glucocorticoid receptor (GR), SCF receptor (c-Kit), as well as BMP4/bone morphogenetic protein receptor (BMPR) signalling in observed effects.

## Materials and methods

### Animals

Male, 6–8 weeks-old CBA mice were obtained from the Breeding Facilities of the Institute for Medical Research, Military Medical Academy, Belgrade. Animals were housed six to eight per cage under conventional conditions (lights on at 06:00 hrs, lights off at 18:00 hrs, 21°C) with standard laboratory diet and water provided *ad libitum*.

The experimental protocol was approved by the Ethic Committee of the Institute for Medical Research, University of Belgrade, Serbia (No. O112-1/12), according to the National Law on Animal Welfare that is consistent with guidelines for animal research and principles of the European Convention for the Protection of Vertebrate Animals Used for Experimental and Other Purposes (Official Daily No. L 358/1-358/6, 18 December 1986) and Directive on the protection of animals used for scientific purposes (Directive 2010/63/EU of the European Parliament and of the Council, 22 September 2010).

### Restraint stress procedure

Mice were individually placed in 50 ml conical centrifuge tubes with multiple ventilation holes, for 2 hrs per day. Restrained mice were maintained horizontally in their home cages during the restraint sessions and released into the same cage thereafter. Animals were subjected to randomly timed (between 07:00 and 11:00 hrs) restraint for 7 or 14 consecutive days. Control mice were handled once daily in the housing room. The animals were killed by cervical dislocation.

### Corticosterone determination

Plasma corticosterone levels were determined using a ^125^I-coupled double antibody radioimmunoassay (MP Biomedicals, LLC, Solon, OH, USA). Corticosterone concentration was expressed as ng/ml.

### Erythropoietin levels

Plasma EPO level was measured using a commercially available ELISA kit (Quantikine; R&D Systems, Minneapolis, MN, USA) according to the manufacturer’s instructions. All samples were individually tested in duplicate and data were depicted as average EPO levels in pg/ml for each group.

### Haematological parameters and iron status

Whole blood was collected from control and restrained mice, and blood cell counts were made using a haemocytometer. Haemoglobin was analysed with the cyanmethemoglobin method using Drabkin’s solution (0.1% sodium bicarbonate, 0.005% potassium cyanide, and 0.02% potassium ferricyanide). Values were determined spectrophotometrically at 540 nm and calculated relative to a standard curve. Haematocrit was calculated after brief centrifugation of blood samples in heparinized microcapillary tubes. Plasma iron status was assessed using the COBAS Integra 400 plus autoanalyser (Roche, Basel, Switzerland).

### Colony assays

Spleens were harvested under sterile conditions, passed through a wire mash and monodispersed in DMEM (Invitrogen, Carlsbad, CA, USA) supplemented with 5% foetal calf serum (FCS; Invitrogen). Then, 4 × 10^5^/ml nucleated splenocytes were plated in methylcellulose media (StemCell Technologies, Vancouver, BC, Canada) containing either 3 U/ml EPO (MethoCult M3334) or 3 U/ml EPO supplemented with 50 ng/ml SCF, 10 ng/ml IL-3, and 10 ng/ml IL-6 (MethoCult GF M3434). Splenocytes were plated in duplicate in 35 mm tissue culture plates (Sarstedt, Nümbrecht, Germany) and incubated at 37°C in a humidified atmosphere containing 5% CO_2_. Following an incubation period of 7 days in MethoCult GF M3434 medium, burst forming units-erythroid (BFU-Es) were enumerated using an inverted microscope. Colony-forming unit-erythroid (CFU-E)-derived colonies were scored after 2 days of culture in MethoCult M3334.

### Immunohistochemistry

The central part of the spleen was dissected under sterile conditions, fixed in formalin and embedded in paraffin wax. The sections, 3 μm thick, were used for immunohistochemical and routine haematoxylin and eosin staining.

The sections were dewaxed, rehydrated and treated with a 0.3% hydrogen peroxide solution to quench endogenous peroxidase activity. Antigen retrieval was aided by placing the slides in a pre-heated 10 mM citrate buffer (pH 6.0) in a microwave oven for 20 min. Slides were then rinsed in PBS and incubated for 1 hr with rat monoclonal anti-Ter119 (1:400 dilution, cat. no. 550565; BD Pharmingen, San Jose, CA, USA), rabbit polyclonal anti-EPOR (1:100 dilution, cat. no. sc-5624; Santa Cruz Biotechnology, Santa Cruz, CA, USA), rabbit polyclonal anti-c-Kit (1:400 dilution, cat. no. A4502; Dako, Glostrup, Denmark) or mouse monoclonal anti-BMP-4 antibody (1:100 dilution, cat. no. VMA1049; AbCys, SA, France). After a brief wash in PBS, immunostaining was performed with the streptavidin-biotin technique and DAB Substrate/Chromogen System for visualization (Novocastra Peroxidase Detection System kit, Leica Biosystems, Wetzlar, Germany). Control sections in which the primary antibody was omitted were processed in parallel. The nuclei were counterstained with Mayer’s haematoxylin. Immunoreactive cells were analysed and scored using a computer-supported imaging system (analysis Pro 3.1) connected to the light microscope (Olympus AX70, Hamburg, Germany) with an objective magnification of ×40.

### Flow cytometry

Freshly isolated splenocytes were passed through a wire mash and monodispersed in DMEM supplemented with 5% FCS. Thereafter, cells were washed in PBS containing 1 mM EDTA and immunostained for 45 min. at 4°C in PBS/0.2% bovine serum albumin with phycoerythrin (PE)-conjugated antimouse Ter119 (cat. no. 553673; BD Pharmingen) and PE/Cy7-conjugated antimouse CD71 antibody (cat. no. 113812; BioLegend, San Diego, CA, USA). Following a subsequent washing, the cells were re-suspended in 1 ml PBS and analysed using a CyFlow SL flow cytometry system (Partec, Münster, Germany).

### Western blot

The spleens were homogenized in chilled RIPA lysis buffer (50 mM Tris-HCl pH 7.6, 150 mM sodium chloride, 1% Triton x-100, 1% sodium deoxycholate, 0.1% sodium dodecyl sulphate, 2 mM EDTA and 50 mM sodium fluoride) at a ratio of 100 mg of tissue to 1 ml of buffer. A protease inhibitor cocktail (Pierce, Thermo Fisher Scientific, Waltham, MA, USA) and sodium orthovanadate were added to the lysis buffer just prior to use. Lysates were incubated at 4°C for 25 min. and then centrifuged at 15,000 × *g*, 4°C, for 20 min. Protein concentration was determined by the BCA Protein Assay Kit (Pierce, Thermo Fisher Scientific) and the samples were stored at −70°C until analysis.

For Western blotting, equal amounts of protein samples were run on polyacrylamide gels and transferred to nitrocellulose membranes (AppliChem GmbH, Darmstadt, Germany). Membranes were probed with primary antibodies (Santa Cruz Biotechnology) to EPOR (1:2000, cat. no. sc-5624), GR (1:1000, cat. no. sc-1004), and actin (1:500, cat. no. sc-1616). Peroxidase-conjugated goat antirabbit immunoglobulin (Santa Cruz Biotechnology) and goat antimouse immunoglobulin (Thermo scientific Pierce), diluted 1:10,000, were used as a secondary antibodies. Western blots were developed using the enhanced chemiluminescence reagent system (GE Healthcare, Amersham, UK) according to the manufacturer’s instructions. The content of EPOR or GR in the tissue extracts was estimated by the densitometry of scanned immunoblot bands using the Image Master Total Lab (GE Healthcare) software.

### Quantitative RT-PCR

Total RNA was isolated from homogenized spleen samples with the RNeasy Mini kit (Qiagen, Manchester, UK) and treated with DNase I (Thermo Fisher Scientific) to remove residual genomic DNA. Equal amounts of RNA from the different samples were transcribed into cDNA using the Maxima First Strand cDNA Synthesis kit (Thermo Fisher Scientific) according to the manufacturer’s instructions. Real-time quantitative PCR was performed on a Mastercycler EP RealPlex (Eppendorf AG, Hamburg, Germany) using the Maxima SYBR Green/ROX qPCR master mix (Thermo Scientific) and the following oligonucleotide primers: BMPR-Ia (forward): 5′-GGGTGTATGAAGTATGAAGGCTCTGAT-3′; BMPR-Ia (reverse): 5′-GCACAAATTGGTCCGACAACATTCTA-3′; BMPR-II (forward): 5′-GACAGAAGTTGGAAACCATCCCACAT-3′; BMPR-II (reverse): 5′-GTGACCTCACTGCCAGGCTATT-3′; BMP4 (forward): 5′-AGGGATCTTTACCGGCTCCA-3′; BMP4 (reverse): 5′-TCCAGATGTTCTTCGTGATGG-3′; glyceraldehyde 3-phosphate dehydrogenase (GAPDH) (forward): 5′-GCACAGTCAAGGCCGAGAAT-3′; GAPDH (reverse): 5′-GCCTTCTCCATGGTGGTGAA-3′. PCR reactions were started with an initial denaturation at 95°C for 8 min., followed by 40 cycles each consisting of denaturation at 95°C for 30 sec., annealing at 60°C for 30 sec., and extension at 72°C for 30 sec. A melting curve analysis was performed after amplification was completed. The relative expression of BMPR-Ia, BMPR-II and BMP4 genes was quantified using the ΔΔCt method normalized to the expression of GAPDH gene.

### Statistical analysis

Data are expressed as mean ± SEM of each group. Statistical significance was assessed by anova. *Post hoc* comparisons using Bonferroni-corrected *t*-test, Fisher’s least significant difference test or Games–Howell test were performed as appropriate. Quantitative RT-PCR analysis was carried out with the Relative Expression Software Tool (REST) 2009 using the pairwise fixed randomization test 21. *P* < 0.05 was considered statistically significant.

## Results

### Chronic restraint stress increases circulating levels of corticosterone and erythropoietin

**Figure 1:**
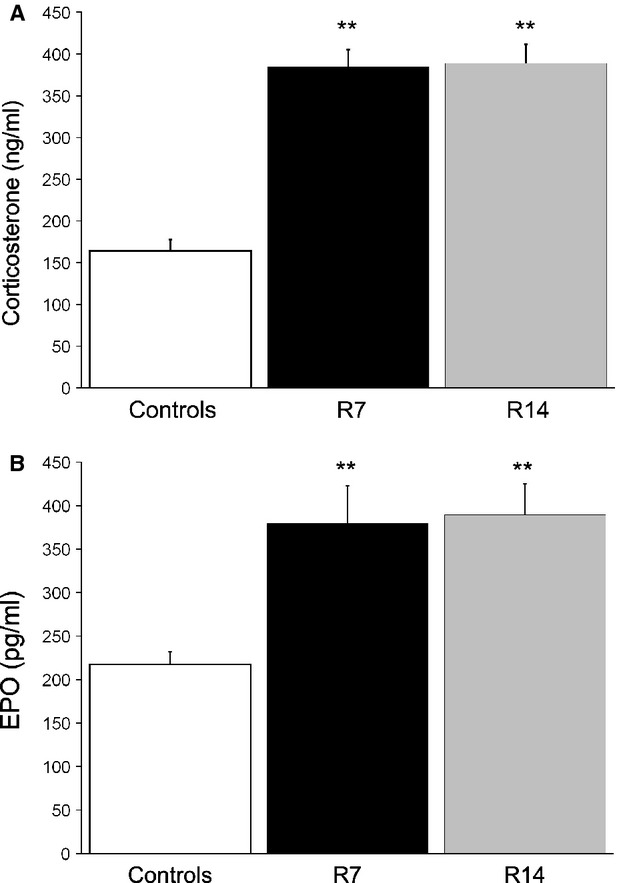
The effects of chronic restraint stress on corticosterone and erythropoietin (EPO) levels. Values are expressed as mean ± SEM;*n* = 10 animals per group. Chronic restraint stress significantly increased plasma levels of corticosterone (A) and EPO (B). Statistical significance was determined using anova followed by the *post hoc,* the Bonferroni *t*-test. **P < 0.01. Animals stressed for 7 (R7) or 14 days (R14).

Repeated restraint is a chronic psychological stressor that activates the hypothalamic-pituitary-adrenal (HPA) axis, and plasma level of corticosterone is considered as a marker of stress. To verify that our chronic restraint procedure activated the HPA axis in mice, we first determined the concentration of plasma corticosterone in restrained and non-stressed animals. Plasma corticosterone levels were significantly increased (*P* < 0.01) after 7 and 14 days of restraint stress, as compared to control mice (Fig. 1A). To further investigate whether chronic restraint stress affected the concentration of EPO in the circulation, we measured plasma EPO levels in all groups. Analysis of plasma EPO concentration revealed that both 1 and 2 weeks of daily restraint stress caused a significant increase (*P* < 0.01) in circulating EPO levels (Fig. 1B).

### Repeated restraint stress decreases iron and haemoglobin levels in the blood

Although there was no significant change in the number of circulating red blood cells, chronic stress markedly decreased (*P* < 0.001; *P* < 0.01) plasma iron levels and the concentration of haemoglobin in the blood (Table 1).

**Table 1:** Haematological parameters and iron status

	Controls	R7	R14
Erythrocytes (10^12^/l)	5.87 ± 0.3	5.98 ± 0.4	5.49 ± 0.2
Leucocytes(10^9^/l)	7.49 ± 0.4	6.56 ± 1.2	5.64 ± 0.9
Platelets (10^12^/l)	1.59 ± 0.1	1.64 ± 0.1	1.43 ± 0.1
Hb (g/l)	150.00 ± 6.1	100.94 ± 4.2***	100.55 ± 7.1***
Hct (%)	52.91 ± 0.6	50.75 ± 1.2	52.20 ± 0.8
MCV (fl)	86.83 ± 4.4	88.26 ± 5.4	90.00 ± 5.7
MCH (pg)	26.88 ± 0.4	19.98 ± 0.7***	18.97 ± 0.3***
MCHC (g/l)	309.72 ± 5.2	226.70 ± 9.6***	210.57 ± 6.8***
RDW (%)	11.36 ± 0.1	13.86 ± 0.3**	14.20 ± 0.3**
Plasma iron (μmol/l)	49.60 ± 0.9	12.96 ± 2.7***	33.06 ± 2.1**
TIBC (μmol/l)	61.12 ± 0.6	24.16 ± 2.4***	48,12 ± 5.3
TS (%)	81.08 ± 1.8	52.84 ± 9.1*	67.90 ± 4.34

Hb: haemoglobin; Hct: haematocrit; MCV: mean corpuscular volume; MCH: mean corpuscular haemoglobin; MCHC: mean corpuscular haemoglobin concentration; RDW: red cell distribution width; TIBC: total iron-binding capacity; TS: transferrin saturation. Data are expressed as mean ± SEM; *n* = 5–10 animals per group. Differences between groups were assesed by anova and *post hoc* Bonferroni *t*-test or Games–Howell test.

*** *P* < 0.001;

** *P* < 0.01;

* *P* < 0.05. Animals stressed for 7 (R7) or 14 days (R14).

### Chronic restraint stress stimulates erythropoiesis in the spleen

To investigate the effects of chronic psychological stress on splenic erythropoiesis, we compared the number of BFU-E, CFU-E and Ter119-positive cells in control and stressed animals. As Ter119 antigen is expressed on erythroid cells from pro-erythroblast through mature erythrocyte stages, we used immunohistochemistry to score nucleated Ter119-positive cells that represent committed erythropoietic precursors beyond the CFU-E stage, as well as to determine the total number of Ter119-positive cells in the spleen. Moreover, in splenic red pulp we analysed the expression of c-Kit, a SCF receptor that is highly expressed on haematopoietic stem cells as well as on erythroid cells from BFU-E to precursors recognized morphologically as basophilic erythroblasts.

**Figure 2:**
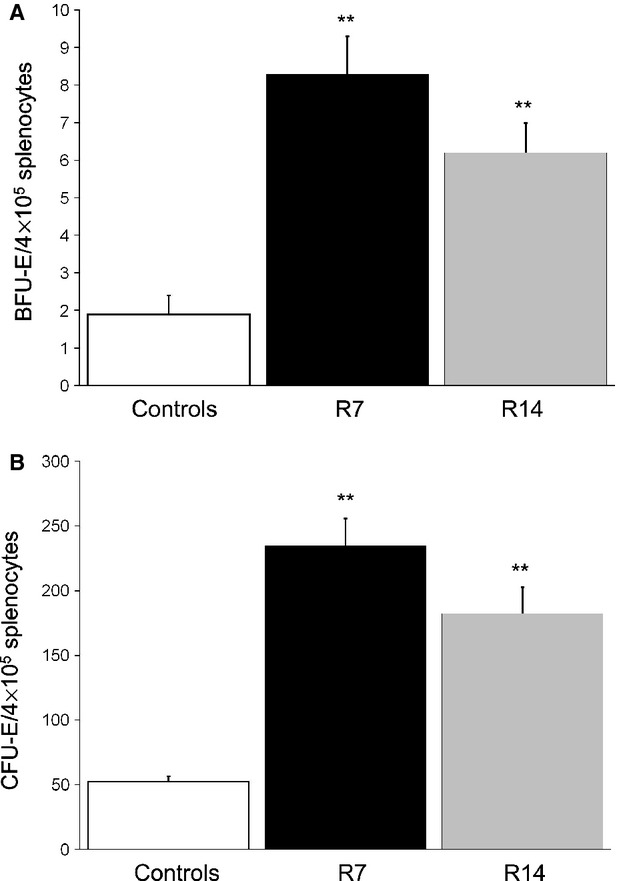
The effects of repeated restraint stress on BFU-E and CFU-E in the spleen. Data are expressed as mean ± SEM;*n* = 8–10 animals per group. Repeated restraint stress significantly increased the number of BFU-E (A) and CFU-E (B). anova followed by the *post hoc,* the Bonferroni *t*-test. **P < 0.01. Animals stressed for 7 (R7) or 14 days (R14).

Chronic exposure to 2 hrs daily restraint stress resulted in markedly increased number of erythroid progenitor cells in the mouse spleen. Compared to untreated controls, there was a significant increase (*P* < 0.01) in both BFU-E and CFU-E progenitors, after either 7- or 14-day period of restraint stress (Fig. 2).

**Figure 3:**
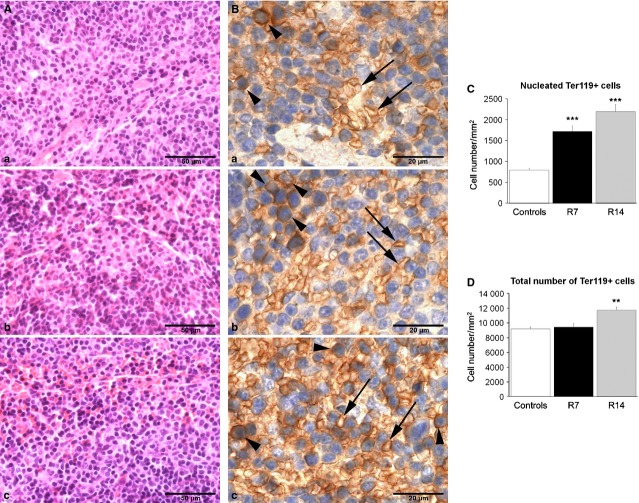
Haematoxylin- and eosin-stained sections and Ter119-positive cells in the red pulp of the spleen. Values for Ter119-positive cells are expressed as mean ± SEM;*n* = 5 animals per group; five high power fields of each tissue section were analysed. Red pulp (A) and Ter119-positive cells (B) in control animal (a), as well as in animals that underwent chronic stress for either 7 (b) or 14 days (c). Arrowheads – nucleated Ter119-positive cells; black arrows – enucleated Ter119-positive cells. Chronic restraint induced an increase in the cellularity of the red pulp (A) as well as a significant increase in the number of nucleated Ter119-positive cells after both 7 and 14 days (B and C), whereas 14 days of restraint also caused the increase in total number of Ter119-positive cells (B and D). anova followed by the *post hoc,* the Bonferroni *t*-test. **P < 0.01; ***P < 0.001. Animals stressed for 7 (R7) or 14 days (R14).

**Figure 4:**
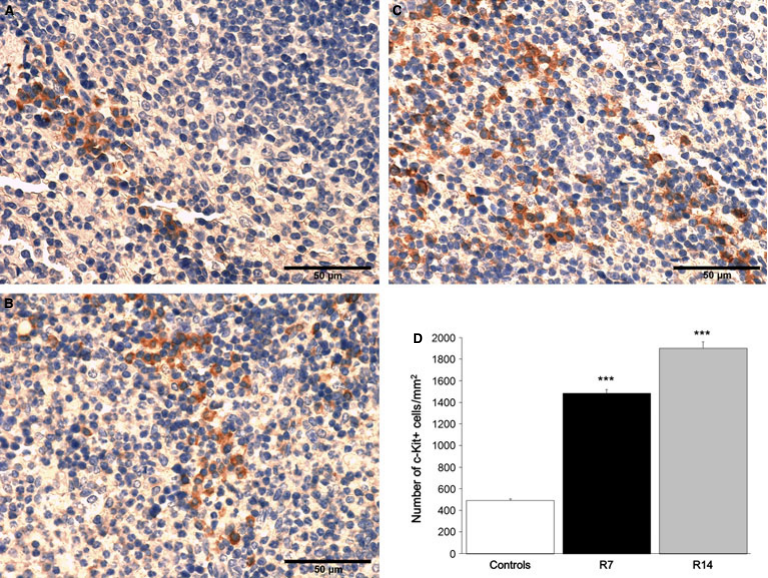
Immunohistochemical analysis of c-Kit-positive cells in the red pulp of the spleen. Data are expressed as mean ± SEM;*n* = 5 animals per group; five high power fields of each tissue section were analysed. c-Kit-positive cells in control animal (A), as well as in animals that underwent chronic stress for either 7 (B) or 14 days (C). Both groups of stressed animals exhibited a significant increase in the number of c-Kit-positive cells as compared to controls (B–D). ***P < 0.001. Animals stressed for 7 (R7) or 14 days (R14).

Morphometric evaluation of the spleen showed a slight increase in volume density of the red pulp in stressed animals as compared to controls, but this increase did not reach statistical significance (Table 2). In addition, histological examination of haematoxylin–eosin-stained sections revealed an increased cellularity of the red pulp in chronically stressed mice (Fig. 3A). Immunohistochemical analysis of Ter119-positive cells in the spleen showed a robust increase (*P* < 0.001) in the number of nucleated Ter119-positive cells after both 7 and 14 days of stress (Fig. 3B and C), whereas 14 days of restraint also caused the increase (*P* < 0.01) in total number of Ter119-positive cells (Fig. 3D). Furthermore, both groups of stressed animals exhibited substantially increased number of c-Kit-positive cells (*P* < 0.001) in the red pulp (Fig. 4).

**Table 2:** Volume densities of major splenic tissue compartments

Vv (%)	Controls	R7	R14
Red pulp	49.50 ± 3.4	52.00 ± 4.9	53.00 ± 3.9
Follicles + PALS	30.60 ± 1.9	29.20 ± 3.2	26.00 ± 2.6
Connective tissue	4.60 ± 0.4	4.40 ± 0.5	3.60 ± 0.5

Vv: volume density; PALS: periarterioral lymphatic sheath. Data are expressed as mean ± SEM; *n* = 5 animals per group. Differences among groups were tested by anova. Animals stressed for 7 (R7) or 14 days (R14).

**Figure 5:**
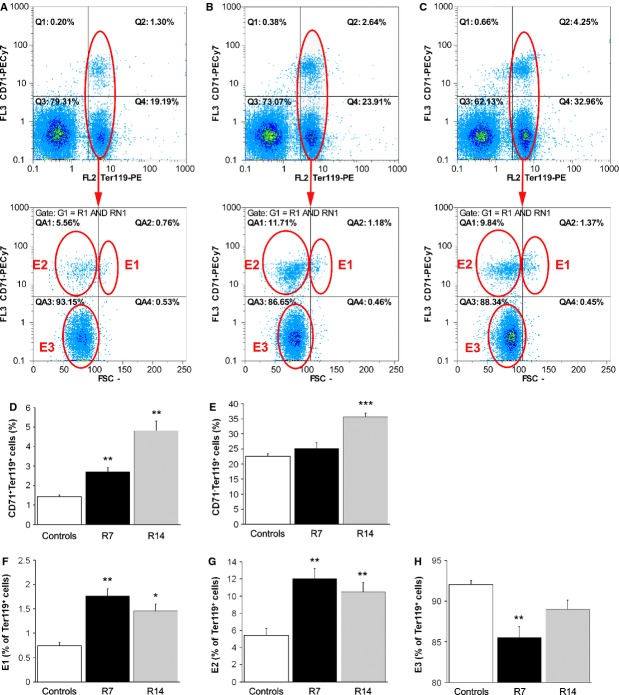
Increased erythropoiesis in the spleens of stressed mice. Representative flow cytometric analysis of splenocytes stained for CD71and Ter119 in control (A), as well as in mice restrained for 7 (B) or 14 consecutive days (C). Values are expressed as mean ± SEM;*n* = 5 animals per group. Chronic restraint induced a significant increase in the percentage of CD71^+^/Ter119^+^ cells after both 7 and 14 days (D) whereas 14 days of restraint also increased the percentage of CD71^−^/Ter119^+^ cells (E). The total Ter119^+^ cells were further analysed for their forward scatter (FCS) and CD71 expression. Repeated restraint increased the percentage of both more (E1) and less (E2) immature Ter119^+^ following 7 and 14 days of stress (F and G), whereas the percentage of the most mature subpopulation (E3) was significantly decreased after 7 days of stress (H). The numbers of CD71^+^/Ter119^+^ and CD71^−^/Ter119^+^ cells are expressed as a percentage of all splenocytes, whereas the numbers of subpopulations at different maturation stages (E, E2 and E3) are expressed as a percentage of Ter119^+^ cells. anova followed by the *post hoc,* the Bonferroni *t*-test. **P < 0.01; ***P < 0.001. Animals stressed for 7 (R7) or 14 days (R14).

In addition, we used flow cytometry to analyse the CD71/Ter119 profile of spleen cells in control (Fig. 5A) and stress-treated (Fig. 5B and C) animals. The exposure to repeated restraint stress for 7 and 14 days elicited a significant increase (*P* < 0.01) in the percentage of CD71/Ter119 double-positive cells (Fig. 5D), whereas 14 days of stress also increased (*P* < 0.001) the percentage of Ter119-single positive cells (Fig. 5E). We subsequently added cell size information to the analysis, in the form of forward scatter parameter, and divided Ter119-positive cells into three subsets according to the maturation stage (Fig. 5A–C). The latter analysis revealed that 7 and 14 days of restraint significantly increased the percentage of both more (E1) and less (E2) immature Ter119-positive cells (Fig. 5F and G). Additionally, the percentage of the most mature subpopulation of Ter119-positive cells (E3) was significantly decreased (*P* < 0.01) in the spleen after 7 days of stress (Fig. 5H).

### Down-regulation of GR and EPOR in the spleen during chronic restraint stress

**Figure 6:**
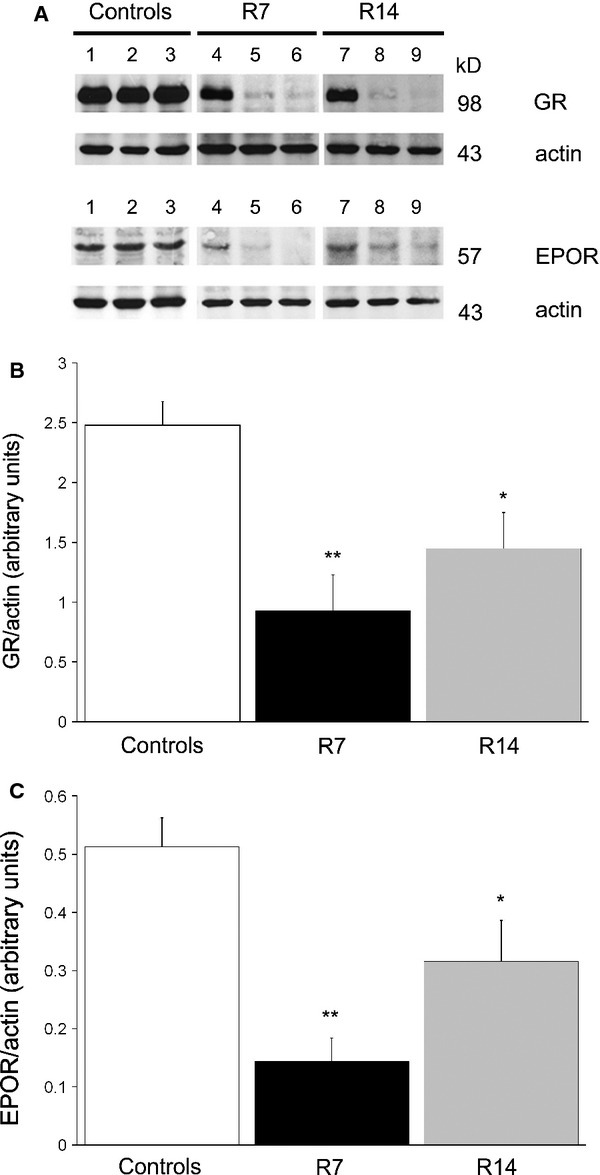
Immunoblotting of GR and EPOR in the spleen. Values are expressed as mean ± SEM;*n* = 7 animals per group. Immunoreactive bands of ∼98 kD for GR and of ∼57 kD for EPOR were detected. Actin was used as loading control. The bands has been additionaly labelled by the numbers. Each number indicates bands, obtained in an individual mouse extract (A). Densitometric analysis of immunoreactive bands revealed that chronic restraint stress significantly decreased expression of both GR (B) and EPOR (C) in the spleen. anova followed by Fisher’s least significant difference *post hoc* test. *P < 0.05; **P < 0.01. Animals stressed for 7 (R7) or 14 days (R14).

**Figure 7:**
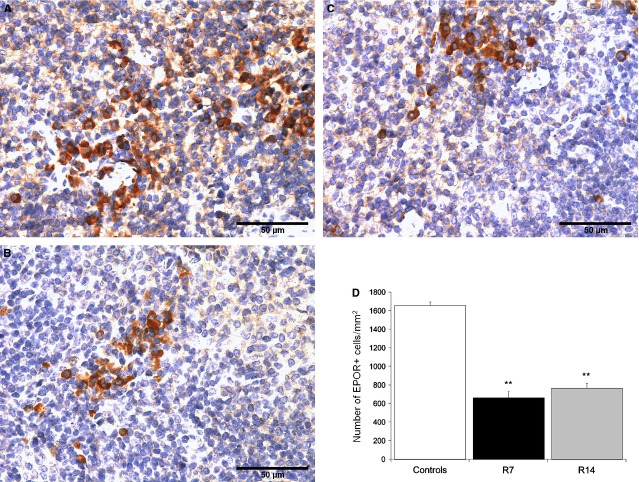
Immunohistochemical analysis of EPOR-positive cells in the red pulp of the spleen. Data are expressed as mean ± SEM;*n* = 5 animals per group; five high power fields of each tissue section were analysed. EPOR-positive cells in control animal (A), as well as in animals that underwent chronic stress for either 7 (B) or 14 days (C). Restrained mice demonstrated a significant decrease in the number of EPOR-positive cells as compared to controls on days 7 and 14 (B–D). anova followed by the *post hoc,* the Bonferroni *t*-test. **P < 0.01. Animals stressed for 7 (R7) or 14 days (R14).

To further investigate potential mediators of these erythropoietic effects induced by chronic psychological stress, we examined the expression of GR and EPOR in the spleen of control and stressed mice. Western blot analysis revealed decreased expression of both receptors in the spleen after 7 (*P* < 0.01) or 14 days (*P* < 0.05) of restraint stress (Fig. 6). Interestingly, comparing the expression levels of these receptors in each animal, it was notable that greater decrease in GR level was accompanied by greater decrease in the level of EPOR and *vice versa* (Fig. 6A). In addition, immunohistochemical analysis showed a down-regulation of EPOR within the red pulp of mice restrained for either 7 or 14 days (Fig. 7).

### BMP4-BMPR signalling is involved in restraint stress-induced splenic erythropoiesis

**Figure 8:**
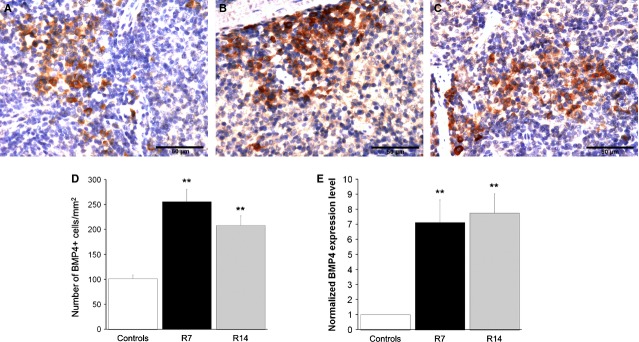
BMP4-positive cells in the red pulp and BMP4 mRNA expression levels in the spleen. Values are expressed as mean ± SEM;*N* = 5 animals per group. BMP4-positive cells in control animal (A), as well as in animals that underwent chronic stress for either 7 (B) or 14 days (C). The number of BMP4-positive cells, determined in whole red pulp, was significantly increased in stressed animals (B–D). anova followed by the *post hoc,* the Games-Howell test. Relative expression of BMP4 mRNA was normalized to GAPDH and reported as fold change compared to untreated controls (E). Differences between groups were determined by Relative Expression Software Tool (REST) 2009 using the pairwise fixed randomization test. **P < 0.01. Animals stressed for 7 (R7) or 14 days (R14).

**Figure 9:**
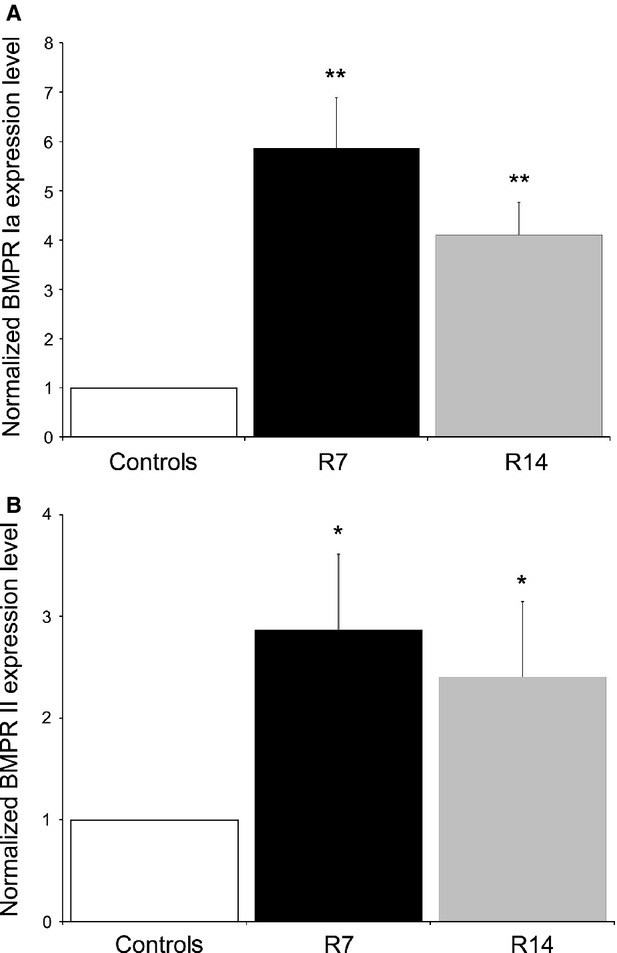
mRNA expression levels of BMPR Ia and BMPR II in the spleen of restrained animals. Relative expression of BMPR Ia mRNA (A) and BMPR II mRNA (B) were normalized to GAPDH, and reported as fold change compared to untreated controls. Values are expressed as mean ± SEM;*n* = 5 animals per group. Differences between groups were determined by Relative Expression Software Tool (REST) 2009 using the pairwise fixed randomization test. *P < 0.05; **P < 0.01. Animals stressed for 7 (R7) or 14 days (R14).

Finally, we examined whether BMP4-BMPR signalling plays a role in extramedullary erythropoiesis induced by chronic psychological stress. Using immunohistochemistry, the expression of BMP4 was detected in the spleen of both stressed and non-stressed mice (Fig. 8A–C). However, compared to controls, chronic restraint elicited a significant increase in the number of BMP4-immunopositive cells in the red pulp after 1 or 2 weeks of repeated procedure (*P* < 0.01, Fig. 8D). Moreover, we demonstrated enhanced expression of BMP4 at mRNA level by qRT-PCR in the spleen following 7 and 14 days of restraint (Fig. 8E). Furthermore, quantitative real-time PCR analysis revealed that expression levels of both BMPR-Ia and BMPR-II genes were significantly higher in the spleen of restrained animals as compared to controls (Fig. 9).

## Discussion

In addition to the well-recognized activation of the neuroendocrine and immune systems, this study demonstrates for the first time that chronic psychological stress stimulates extramedullary erythropoiesis in the spleen. Moreover, we show that exposure to chronic psychological stress enhances spleen erythropoiesis through the involvement of GR, EPOR, c-kit and BMP4, the key mediators of SE, previously described in animal models of anaemia.

Psychological stress is increasingly becoming an important aspect of daily life 22 and understanding its biological implications requires an integrative approach. Restraint stress, which acts primarily as a psychological stressor, has an inhibitory influence on myeloid progenitors in the bone marrow 6. However, there are very limited data regarding the effects of chronic psychological stress on erythropoiesis in adult mammals 23. Thus, Sherstoboev and Minakova reported an expansion of erythroid progenitor cells in the bone marrow along with an increased serum erythropoietic activity during chronic immobilization stress 7. Although our unpublished results indicate that chronic restraint stress also exerts a stimulatory effect on erythroid progenitors in the bone marrow, we have focused on spleen as a major site of erythropoiesis under different stress conditions 8.

Acute anaemic stress induces a physiological response that includes the complete mobilization and rapid expansion of erythroid progenitors in the spleen 18. Furthermore, Millot *et al*. used murine model of chronic inflammation leading to long-term anaemia, and demonstrated that treatment with EPO did not increase bone marrow erythropoiesis, but rather induced SE in the spleen 24. Accordingly, our results showed that chronic exposure to daily restraint stress decreased the concentration of haemoglobin in the blood, elevated circulating EPO levels, and caused a marked increase in both BFU-E and CFU-E progenitors in the mouse spleen. In addition, there is a growing evidence indicating that erythroid progenitors in the spleen are distinct from those in the bone marrow and supporting the existence of ‘stress BFU-E’, a specialized population of progenitor cells that are resident in the spleen 8,13. Consistent with this, we found that, unlike bone marrow BFU-E, which required both EPO and a burst-promoting signal, spleen BFU-E from chronically stressed animals formed colonies in media containing only EPO (data not shown). This observation is similar to human foetal BFU-E 18–25, endorsing the assumption that expansive erythropoiesis in the adult spleen corresponds to that observed during foetal development 26.

In addition to increased number of erythroid progenitors, chronic restraint stress also caused a significant increase in the number of more mature erythroid cells – nucleated Ter119-positive cells. However, 7 days of repeated restraint did not increase the total number of Ter119-positive cells (comprised of both nucleated Ter119-positive cells and mature erythrocytes). The total number of Ter119-positive cells was significantly increased only after 14 days of restraint stress, indicating a delay in terminal erythroid differentiation. Delayed terminal differentiation of erythroid cells in stressed animals was further confirmed by flow cytometric analysis of splenocytes stained for cell surface markers CD71 and Ter119. Thus, flow cytometry revealed markedly increased percentage of immature Ter119-positive cells in the spleen following 7 and 14 days of restraint, whereas the percentage of the most mature subpopulation of Ter119-positive cells was significantly decreased after 7 days of stress. Likewise, using flow cytometric analysis, Liu *et al*. showed a dramatic increase in the frequency of splenic early erythroblasts during the erythropoietic stress response *in vivo* 27.

Further analysis of the erythropoietic stress response has demonstrated the involvement of GR and EPOR in this process 28–29. Accordingly, GR was found to have a key role in the regulation of erythroid progenitor self-renewal 30. The observation that polycythemia is the first manifestation of Cushing’s disease 31, a syndrome associated with chronic stimulation of GR, provides an additional evidence for this finding. Subsequently, von Lindern *et al*. showed that GR co-operates with the EPOR to enhance the proliferation of erythroid progenitors *in vitro* 32. Furthermore, Stellacci *et al*. demonstrated a direct interaction between GR and EPOR through their physical association in the membrane of human erythroid cells, suggesting a membrane-associated pathway of GR and EPOR signalling 33. In agreement with the results showing that chronic restraint stress down-regulates the expression of GR in the pre-frontal cortex 34, we have observed decreased expression of GR in the spleen of chronically restrained mice. Thus, decreased expression of GR and EPOR in the spleen along with an increase in the circulating levels of glucocorticoids and EPO, observed in restrained mice, indicate a ligand-induced activation of these receptors under chronic stress. Moreover, analysing the expression level in each animal, we found that greater decrease in GR was accompanied by greater decrease in EPOR, and *vice versa*. This finding may indicate a physical association between GR and EPOR during their activation, and, therefore, might provide the first *in vivo* evidence for their direct interaction.

Unravelling the mechanisms involved in anaemia-induced stress response, Perry *et al*. have reported that stress BFU-E cells express c-Kit on their surface 11. In accordance, the increased number of c-Kit-positive cells in the red pulp, observed in this study, may reflect an increased population of stress BFU-E cells in the spleen of chronically stressed mice. A profound insight into the erythropoietic role of SCF/c-Kit signalling was additionally provided by the development of mutant mouse models. Thus, it has been found that mice mutant for the c-Kit receptor is slow to recover from acute anaemia 11–35. Delayed kinetics of recovery from anaemia has also been demonstrated in splenectomized mice, as compared to controls 12. Moreover, the bone marrow of splenectomized mice did not compensate for this delay by increasing erythropoiesis. Although the role of spleen in human SE still remain to be elucidated, our results together with previously published studies 14–24 underscore the importance of the spleen in this process, especially considering that bone marrow cannot simply increase erythropoietic rate on a relatively massive scale 12.

An essential role of the spleen in stress-induced erythropoiesis is further supported by the fact that its microenvironment contains a signal which promotes the development of stress erythroid progenitors. Recently, Perry *et al*. have identified Hedgehog and BMP4 as specific signals in the spleen that are required for SE pathway 36. Hedgehog signalling in the spleen induces the expression of BMP4, a signal that has a crucial role for the rapid expansion of stress erythroid porgenitors. BMP4 binds to heteromeric receptor complexes composed of type I and type II transmembrane receptors (BMPR), thus initiating a signalling pathway that plays an important role during organ development and tissue regeneration 37. Consistent with its role in mammalian development and tissue homeostasis, BMP4 is normally expressed in a wide range of foetal tissues, as well as in several adult tissues including the splenic red pulp 38. Concordantly, we have detected BMP4-immunopositive cells in the spleen of both control and chronically stressed mice. However, exposure to chronic restraint stress markedly increased the number of BMP4-positive cells in splenic red pulp and also caused a notable increase in splenic BMP4 mRNA expression level. The elevated expression levels of mRNA for both BMPR-Ia and BMPR-II, together with an increased BMP4 gene and protein expression in the spleen of restrained animals, indicate the involvement of BMP4/BMPR signalling pathway in the erythropoietic response to chronic psychological stress.

Even though an analogous pathway has not yet been identified in humans, few studies support a connection between the murine BMP4-dependent extramedullary erythropoiesis and SE in humans 18. Furthermore, human SE, described during recovery from bone marrow transplantation as well as from transient erythroblastopenia, exhibits some properties of foetal erythropoiesis, such as the expression of foetal red blood cells antigens and foetal haemoglobin 18–39.

We also demonstrated that exposure to restraint for 7 and 14 days significantly decreased plasma iron concentration. Our data, consistent with previous studies in chronically stressed animals 23–40, clearly indicate that reduced amount of haemoglobin in restrained mice is the result of low level of circulating iron. In accordance, Chen *et al*. have showed that chronic psychological stress reduces the absorption of iron, leading to subsequent iron deficiency 41. Moreover, iron deficiency itself is associated with an increased rate of erythropoiesis and a prevalence of erythroid precursors that become quiescent or proliferate without completing the maturation cycle 42. Similarly, in leukaemia, progenitors derived from both pluripotent stem cells and multi- or unipotent progenitors can undergo long-term proliferation usually without entering terminal differentiation 43. Prolonged activation of SE pathways as well as delayed terminal erythroid differentiation, observed in this study, may represent a potential risk for leukaemic transformation. Additionally, the spleen has been recognized as a microenvironment that accelerates the expansion of erythroleukaemic cells 44. The fact that acute leukaemic transformation in myeloproliferative neoplasms can occur in the spleen 10 further support a role of splenic microenvironment in creating conditions favourable to leukaemic transformation.

In conclusion, our results show that chronic psychological stress decreases haemoglobin levels in the blood and activates BMP4-dependent extramedullary erythropoiesis, leading to the robust expansion of erythroid progenitors and precursors in the mouse spleen. Prolonged activation of SE pathways along with an excessive production of immature erythroid cells during chronic psychological stress may predispose chronically stressed subjects to a higher risk of leukaemic transformation. Further studies are needed to examine a connection between low haemoglobin levels in the blood and an enhanced erythropoiesis in the spleen, as well as to unravel the molecular background underlying the erythropoietic response to chronic psychological stress.
